# Associations of plaque morphology and location with Intraplaque neovascularization in the carotid artery by contrast-enhanced ultrasound imaging

**DOI:** 10.3389/fneur.2023.1097070

**Published:** 2023-05-11

**Authors:** Shi-Yao Gu, Lu-Ni Zhang, Jing Chen, Fang Li, Ming-Hua Yao, Cai-Xia Jia, Rong Wu

**Affiliations:** Department of Sonography, The General Hospital Affiliated to Shanghai Jiaotong University, Hongkou, Shanghai, China

**Keywords:** carotid artery, plaque, atherosclerotic, contrast-enhanced ultrasound, neovascularization

## Abstract

**Objective:**

Intraplaque neovascularization (IPN) is a known indicator of plaque vulnerability, and is thus considered a predictor of stroke. The morphology and location of the carotid plaque may be correlated with plaque vulnerability. Therefore, our study aimed to examine the associations of carotid plaque morphology and location with IPN.

**Methods:**

A total of 141 patients with carotid atherosclerosis (mean age, 64.99 ± 10.96 years) who underwent carotid contrast-enhanced ultrasound (CEUS) between November 2021 and March 2022 were retrospectively analyzed. IPN was graded according to the presence and location of microbubbles within the plaque. The association of IPN grade with carotid plaque morphology and location was evaluated using ordered logistic regression.

**Results:**

Of the 171 plaques, 89 (52%) were IPN Grade 0, 21 (12.2%) were Grade 1, and 61 (35.6%) were Grade 2. IPN grade significantly associated with both plaque morphology and location, with higher grades observed among Type III morphology and common carotid artery plaques. Significant negative association was further shown between IPN grade and serum high-density lipoprotein cholesterol (HDL-C) level. Plaque morphology and location, and HDL-C remained significantly associated with IPN grade after adjusting for confounding factors.

**Conclusion:**

The location and morphology of carotid plaques were significantly associated with the IPN grade on CEUS, and therefore show potential as biomarkers for plaque vulnerability. Serum HDL-C was also identified as a protective factor against IPN, and may play a role in the management of carotid atherosclerosis. Our study provided a potential strategy for identification of vulnerable carotid plaques and elucidated the important imaging predictors of stroke.

## 1. Introduction

Stroke, with its high morbidity, mortality, and disability rates, represents one of the leading causes of death worldwide (1). Approximately 18–25% of all ischemic strokes are attributable to carotid plaque rupture (2, 3). Intraplaque neovascularization (IPN) represents an important feature of plaque vulnerability (4). IPN provides valuable insight to plaque activity, as it has been reported to associate with an increased risk of neovessel rupture, intraplaque hemorrhage, and inflammation (5).

The location and morphology of plaques have been shown to contribute to plaque vulnerability (6). The potential mechanism for this has been reported to relate to their influence on the shear stress generated on plaque surfaces (7), which is a known key player in the pathophysiology of atherosclerosis (8, 9). Indeed, areas of low shear stress are often accompanied by higher expressions of inflammatory mediators and greater degree of matrix metalloproteinase activity (10). Moreover, inflammation is known to initiate the process of neovascularization. However, the exact influence of plaque location and morphology on the degree of IPN remains unknown.

Contrast enhanced ultrasound (CEUS), a novel ultrasound technique, has been recognized as an effective imaging modality for detecting neovascularization (11, 12). Intensity of plaque enhancement on CEUS has been shown to significantly correlate with the degree of neovascularization (13–15). In addition, CEUS has allowed for clearer visualization of both the location and morphological features of carotid plaques as compared to standard duplex ultrasound.

As such, our study aimed to evaluate the association of plaque location and morphology with IPN grade on CEUS, to assess their role as potential biomarkers for plaque vulnerability.

## 2. Materials and methods

### 2.1. Study population

**Figure 1:**
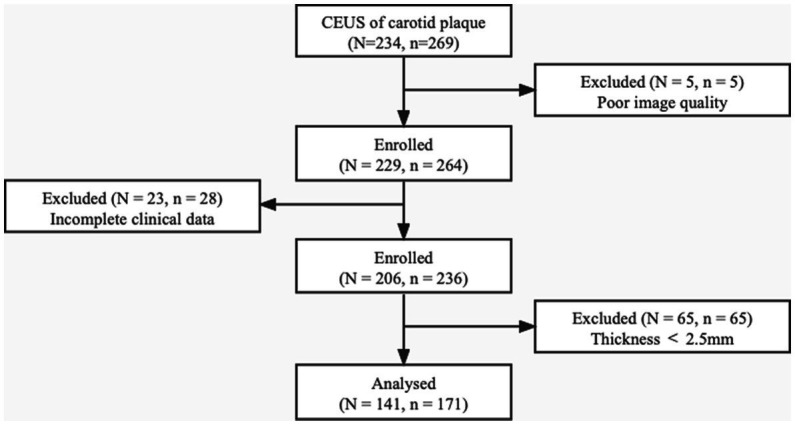
The patient selection process. Among the 234 patients diagnosed with carotid plaques, 93 (98 plaques) were excluded for poor image quality, incomplete clinical data, and plaque thickness < 2.5 mm. *N*, number of patients; *n*, number of plaques.

Consecutive patients diagnosed with carotid atherosclerotic plaques who underwent CEUS between November 2021 and March 2022 were retrospectively analyzed. The inclusion criteria involved carotid plaques of thickness ≥ 2.5 mm measured in the longitudinal axis at the point of greatest luminal narrowing. This was selected based on guideline reports that plaques of such size group are clinically significant, and can be accurately assessed on ultrasound (16). The exclusion criteria included: (1) maximum plaque thickness < 2.5 mm; (2) poor image quality such as severe plaque calcification; (3) allergy to CEUS contrast agent; (4) severe cardiopulmonary dysfunction or intolerance to CEUS; and (5) incomplete clinical data (Figure 1).

### 2.2. Clinical variables

The following variables were collected: (1) age, sex, and body mass index (BMI); (2) medical history, including hypertension, diabetes, and coronary artery disease; (3) smoking history; (4) statin use; and (5) blood test results, including low density lipoprotein cholesterol (LDL-C), high density lipoprotein cholesterol (HDL-C), total cholesterol (TC) and triglyceride (TG) levels.

### 2.3. Morphology and location of carotid plaques

The morphology and location of plaques were evaluated using combined B-mode ultrasound and CEUS.

**Figure 2:**
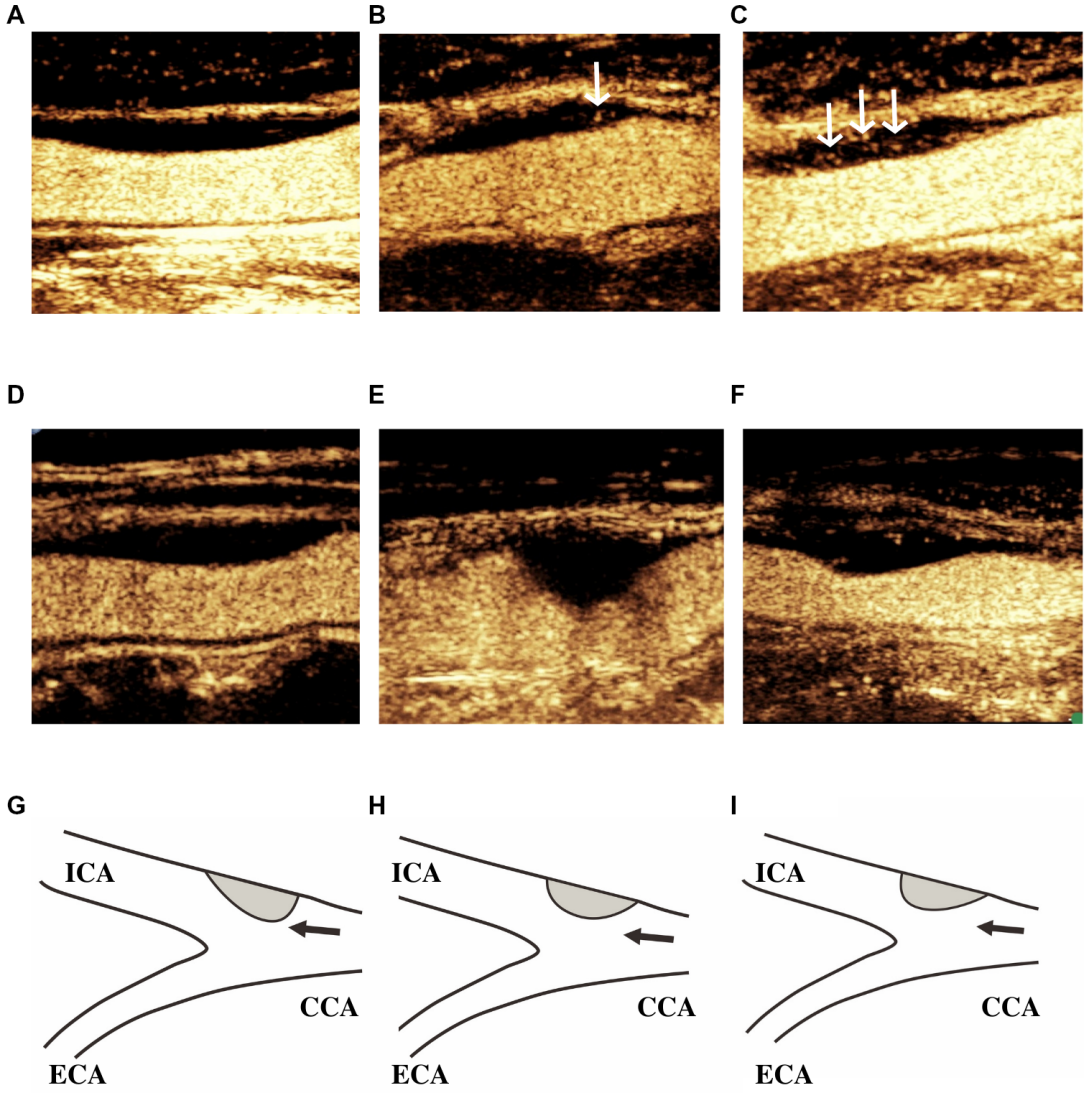
IPN grade and carotid plaque morphology. Classification of IPN grade: **(A)** Grade 0, no bubbles within the plaque, or bubbles confined to the adventitial side; **(B)** Grade 1, moderate intraplaque enhancement with moving bubbles at the adventitial side in the plaque shoulder; and **(C)** Grade 2, extensive intraplaque enhancement with clear appearance of bubbles moving into the plaque core. White arrows indicate intraplaque enhancement. Classification of plaque morphology: **(D,G)** Type I, the greater arc-length of the carotid plaques was located in the downstream arterial wall above the site showing maximum wall thickness; **(E,H)** Type II, the arc-lengths of the carotid plaques in the downstream and upstream arterial walls from the site showing maximum wall thickness were equal; and **(F,I)** Type III, the greater arc-length of carotid plaques was located in the upstream arterial wall below the site showing maximum wall thickness. Black arrows indicate the direction of blood flow. ICA, internal carotid artery; ECA, external carotid artery; and CCA, common carotid artery.

Plaque morphology was assessed in terms of symmetric features in the longitudinal axis. Arc length was measured as the distance from each end of the plaque to point of maximum thickness. The morphology was classified as Type I (the greater arc-length of the carotid plaque was located in the downstream arterial wall above the site with maximum wall thickness), Type II (the arc-lengths of the carotid plaques in the downstream and upstream arterial walls from the site with maximum wall thickness were equal, and the tolerances were no less than 1 mm), or Type III (the greater arc-length of the carotid plaque was located in the upstream arterial wall below the site showing maximum wall thickness; Figure 2) (6).

Plaque location was divided into the internal carotid artery (ICA), carotid bifurcation, and common carotid artery (CCA). In the case of plaques spanning across two locations, the position of the point of maximum thickness was considered.

In addition, we recorded the presence of ulceration (cavities measuring at least 1 mm), which has been associated with the risk of plaque rupture (4, 17).

### 2.4. B-mode and contrast-enhanced ultrasound

All B-mode ultrasound and CEUS examinations were performed by an experienced radiologist using Philips EPIQ Elite (Philips, Netherlands) with a high frequency probe (eL18-4, MHz). After identification of the target plaque, the maximum longitudinal view of the plaque was determined for CEUS analysis.

CEUS was performed after bolus injection of 1.0 ml SonoVue solution (Bracco, Milan, Italy) followed by 5 ml saline flushing through a peripheral vein. The system settings were as follows: mechanical index, 0.06; gain, 60 ~ 70%; and depth, 2.5–3.5 cm. During the examination, the patients were encouraged to maintain calm breathing, and to avoid swallowing or coughing as best as possible.

Following contrast agent administration, IPN was graded using a semiquantitative visual approach according to the presence and location of microbubbles in the plaque. IPN grading was as follows: Grade 0 (no bubbles within the plaque, or bubbles confined to the adventitial side), Grade 1 (moderate intraplaque enhancement with moving bubbles at the adventitial side in the plaque shoulder), and Grade 2 (extensive intraplaque enhancement with clear appearance of bubbles moving into the plaque core; Figure 2) (18). All videos (of at least 2 min in duration) were stored digitally on magnetic optical disks for offline analysis.

Inter-observer consistency in IPN grading was analyzed by two independent radiologists (S-YG and L-NZ) who were blinded to each other’s interpretation. To evaluate intra-observer consistency, the data was reanalyzed by the same radiologist (S-YG) after an interval of 1 month without reference to the initial results.

### 2.5. Statistical analysis

All statistical analyzes were performed using SPSS 25.0 (IBM, Armonk, NY, United States). Categorical and continuous variables were expressed as frequency (%) and mean ± standard deviation (SD), respectively. Analysis of variance was performed to compare the characteristics of both patient and plaque based on IPN grade. Ordered logistic regression analysis was used to analyze the relationship of IPN grade with selected factors after adjusting for confounding factors, with outcomes expressed as odds ratio (OR) and 95% confidence interval (CI). Intra- and inter-observer consistencies were analyzed using the intra-group correlation coefficient. Statistical significance was considered as *p* < 0.05.

## 3. Results

### 3.1. Baseline characteristics

Among the 234 patients who underwent carotid artery CEUS, 93 were excluded due to poor image quality (N = 5), incomplete clinical data (*N* = 23), and plaque thickness < 2.5 mm (*N* = 65; Figure 1). A total of 141 patients were eventually enrolled, of whom 107 (75.9%) were male. The average age was 64.99 ± 10.96 years. In terms of clinical characteristics, 52 (36.9%) had diabetes, 79 (56.0%) had hypertension, 20 (14.2%) had coronary heart disease, 42 (29.8%) had positive statin use history, and 74 (52.5%) had positive smoking history. All baseline characteristics of the included patients are presented in Table 1.

**Table 1:** Baseline clinical characteristics of the included patients.

Clinical characteristic	Patients (*n* = 141)
Male sex, *n* (%)	107 (75.9)
Age (year)	64.99 ± 10.96
BMI (kg/m^2^)	24.85 ± 4.81
Hypertension, *n* (%)	79 (56.0)
Diabetes, *n* (%)	52 (36.9)
Coronary heart disease, *n* (%)	20 (14.2)
LDL-C, mmol/L	2.81 ± 0.84
HDL-C, mmol/L	1.27 ± 0.37
TC, mmol/L	4.75 ± 1.17
TG, mmol/L	1.46 ± 0.97
Smoking history	74 (52.5)
Statin use history	42 (29.8)

BMI, body mass index; LDL-C, low density lipoprotein cholesterol; HDL-C, high density lipoprotein cholesterol; TC, total cholesterol; and TG, triglyceride.

Bilateral carotid CEUS was performed in 30 (21.3%) patients. Among a total of 171 carotid plaques, 89 (51.4%) were Grade 0, 21 (12.1%) were Grade 1 and 63 (36.4%) were Grade 2. The comparison of patient and ultrasound characteristics based on IPN grade are shown in Tables 2, 3, respectively.

**Table 2:** Comparison of clinical characteristics based on IPN grade.

	Grade 0 (*n* = 89)	Grade 1 (*n* = 21)	Grade 2 (*n* = 61)	OR	95% CI	*p*
Male, *n*	64	17	49	1.56	0.79–3.11	0.21
Age, year	65.30 ± 10.18	65.48 ± 10.61	65.61 ± 11.82	1.00	0.98–1.03	0.86
BMI, kg/m^2^	24.19 ± 3.89	25.35 ± 6.47	25.12 ± 5.08	1.04	0.97–1.10	0.25
Hypertension, *n*	50	11	41	0.69	0.38–1.24	0.21
Diabetes, *n*	32	10	21	1.02	0.56–1.85	0.95
Coronary heart disease, *n*	12	4	8	0.99	0.43–2.27	0.99
LDL-C, mmol/L	2.94 ± 0.77	2.80 ± 1.01	2.85 ± 0.89	0.89	0.63–1.26	0.52
HDL-C, mmol/L	1.31 ± 0.40	1.17 ± 0.32	1.17 ± 0.29	0.33	0.13–0.81	0.02^*^
TC, mmol/L	4.78 ± 1.15	4.51 ± 1.30	4.62 ± 1.18	0.90	0.70–1.15	0.39
TG, mmol/L	1.39 ± 0.91	1.31 ± 0.43	1.55 ± 1.00	1.20	0.86–1.68	0.28
Statin use history	23	7	19	1.06	0.59–1.88	0.85
Smoking history	47	12	31	0.78	0.42–1.48	0.45

BMI, body mass index; LDL-C, low density lipoprotein cholesterol; HDL-C, high density lipoprotein cholesterol; TC, total cholesterol; and TG, triglyceride; CCA, common carotid artery; ICA, internal carotid artery.

**Table 3:** Comparison of ultrasound characteristics based on IPN grade.

	Grade 0 (*n* = 89)	Grade 1 (*n* = 21)	Grade 2 (*n* = 61)	OR	95% CI	*p*
Echoes, hypoechoic	66	11	39	1.26	0.92–1.71	0.14
Ulceration	6 (4.5%)	1 (4.8%)	3 (6.6%)	1.38	0.39–4.87	0.62
Maximum thickness	3.22 ± 0.77	3.41 ± 1.08	3.35 ± 0.82	1.19	0.84–1.68	0.34
Carotid plaque morphology						
Type I	47	6	14	2.06	1.47–2.90	<0.01^*^
Type II	17	3	10			
Type III	25	12	37			
Carotid plaque location						
CCA	14	3	20	1.30	1.02–1.65	0.04^*^
Carotid bifurcation	59	15	34			
ICA	16	3	7			

### 3.2. Relationship between carotid plaque morphology and IPN grade

IPN grade was observed to significantly associate with plaque morphology (OR, 2.06; 95% CI, 1.47–2.90; *p* < 0.01; Table 3), with higher IPN grades observed among Type III plaques. Significant differences in IPN grade were observed between Type I and III plaques, as well as between Type II and III plaques (*p* < 0.01 and *p* = 0.04, respectively). However, no significant differences were demonstrated between Type I and II plaques (*p* = 0.18).

### 3.3. Relationship between carotid plaque location and IPN grade

Plaques located in the CCA demonstrated significantly higher IPN grades (OR, 1.30; 95% CI, 1.02–1.65; *p* = 0.04; Table 3). Significant differences in IPN grades were observed between plaques located in the CCA and the carotid bifurcation (*p* = 0.03), as well as between those in the CCA and the ICA (*p* = 0.03). However, no significant differences were shown between plaques located in the carotid bifurcation and the ICA (*p* = 0.57).

### 3.4. Relationship between serum HDL-C level and IPN grade

Lower serum HDL-C level was observed to significantly associate with higher IPN grade (OR, 0.33; 95% CI, 0.13–0.81; *p* = 0.02; Table 2).

### 3.5. Logistic regression analysis

After adjusting for confounding factors such as gender, BMI, hypertension, smoking history, and statin use, all 3 factors remained statistically significant. Significantly higher IPN grades were demonstrated among plaques of Type III morphology (OR, 2.09; 95%CI, 1.48–2.96; *p* < 0.01) and those located in the CCA (OR, 1.37; 95%CI, 1.04–1.77; *p* = 0.02). In contrast, a significant negative association was shown between serum HDL-C level and IPN grade (OR, 0.27; 95%CI, 0.10–0.76; *p* = 0.01; Table 4).

**Table 4:** Logistic regression analysis.

	IPN grade								
	Univariate regression			Model 1			Model 2		
	OR	95% CI	*p*	OR	95% CI	*p*	OR	95% CI	*p*
Plaque morphology	2.06	1.47–2.90	<0.01^*^	2.09	1.48–2.96	<0.01^*^	2.25	1.55–3.27	<0.01^*^
Plaque location	1.30	1.02–1.65	0.04^*^	1.37	1.04–1.77	0.02^*^	1.36	1.04–1.77	0.02^*^
HDL-C	0.33	0.13–0.81	0.02^*^	0.29	0.11–0.83	0.02^*^	0.27	0.10–0.73	0.01^*^

Model 1, adjusted for gender, BMI. hypertension, smoking history, and statin use.

Model 2, adjusted for gender, BMI. hypertension, smoking history, statin use, echo, maximum thickness, and ulceration.

After adjusting for echo, maximum thickness, and ulceration, plaque morphology (OR, 2.25; 95%CI, 1.55–3.27; *p* < 0.01), plaque location (OR, 1.36; 95%CI, 1.04–1.77; *p* = 0.02), and serum HDL-C (OR, 0.27; 95%CI, 0.10–0.73; *p* = 0.01) remained statistically significant (Table 4).

### 3.6. Relationship of plaque morphology and location with ulceration

Ulceration was observed on 11 plaques, but did not demonstrate any significant correlation with morphology or location (*p* = 0.25 and *p* = 0.13, respectively; Supplementary Table S1). No significant correlation was demonstrated with IPN grade as well (*p* = 0.62; Table 3).

### 3.7. Intra- and inter-observer consistency analysis

Excellent agreement in the CEUS evaluation of IPN was demonstrated. The intra-observer consistency was 0.88 (95% CI, 2.32–2.45; *p* < 0.01), while the inter-observer consistency was 0.85 (95% CI, 2.23–2.43; *p* < 0.01).

## 4. Discussion

The morphology and location of carotid plaques, as well as serum HDL-C level, demonstrated a significant influence on IPN grade in our study.

Higher IPN grades were observed among plaques of Type III morphology. Plaques of such morphology are characterized by lower upstream slopes, which may be subjected to lower shear stress (6). This is consistent with the notion that proatherogenic transcription factor upregulation and the resultant aggregation of inflammatory cells tend to occur in regions of low shear stress (19), ultimately resulting in a more fragile plaque phenotype (20, 21). Our findings of increased neovascularization in a low shear stress environment are further in correspondence to previous reports that macrophage infiltration often coexist with hypoxia and angiogenesis due to high metabolic demand (22, 23).

The bifurcation of the CCA has long been known as a common location for atherosclerotic plaque development due to the disturbance in flow (24, 25). However, we found that plaques located in the CCA were associated with a higher IPN grade instead. We postulate that this may be related to the greater length and area of such plaques (Supplementary Table S2; Supplementary Figure S1), which may have reflected greater plaque burden. In line with this, it has been reported that plaques of the CCA tend to grow along the longitudinal axis of the vessel wall and create greater lengths (26). Larger plaques may thereby associate with larger areas of anoxia, greater degrees of inflammation, and ultimately increased neovascularization (26, 27).

Serum HDL-C was found to be significantly protective against neovascularization. While low-density lipoprotein cholesterol (LDL-C) is widely accepted as an independent predictor of cardio- and cerebrovascular events, low HDL-C levels have been reported to be associated with an increased risk of cardiovascular diseases and stroke regardless of LDL-C levels (28–30). The HDL-C level was negatively associated with IPN grading in our study, and this association did not relate to statin use. Other lipid parameters, including the LDL-C level, were not significantly associated with IPN grading, highlighting the potential importance of HDL-C in plaque vulnerability. HDL-C is known to promote the reverse transport of cholesterol from atherosclerotic plaque (31). In addition, HDL-C portrays anti-atherosclerotic effects, which is mediated by its antioxidant, anti-inflammatory, and antithrombotic characteristics (32, 33). While Ying et al. (34) have found an association of carotid plaque neovascularization with total cholesterol and LDL-C levels. This was, however, not observed in our study, which may be attributable to the use of statins (Supplementary Table S3).

Ulceration is an important feature of ruptured plaques (35). However, we found no correlation of ulceration with IPN grade, plaque morphology, or plaque location. This may be due to the relatively few cases of ulcerated plaques among our patients, which hindered effective statistical analysis. However, a previous study showed that shear stress and local hemodynamics caused by anatomical differences of the carotid arteries did not influence the incidence of plaque ulceration (36). This suggests that morphology and location may not play any role in such feature. Nonetheless, further large-sample analyzes are warranted to elucidate the factors involved in the development of ulcerated plaques.

There were several limitations in our study. First, this was a single-centered study with a relatively small sample size. Large-sample studies involving the assessment of other potential associating factors such as clinical symptoms are thereby warranted. Second, plaque morphology was assessed based on only the longitudinal views of B-mode ultrasound and CEUS. However, the spatial morphology of plaques is complicated and may be correlated with plaque vulnerability. In future studies, we aim to evaluate the spatial diversity of plaque morphology through three-dimensional ultrasound imaging. Third, the geometry of calcification, which may affect plaque stability, was overlooked in our study. This was due to their acoustic attenuation effects on CEUS, which would hinder our assessment of neovascularization. Further studies on the effects of calcification on plaque vulnerability are thus required. Finally, plaques with thickness < 2.5 mm were excluded due to the difficulties in performing CEUS for plaques with a thinner wall, which could have negatively affected the accuracy of our results. Some studies on the IPN grade excluded plaques with thickness < 2.5 mm for similar reasons (37). To obtain more accurate results, we excluded these data. In future studies, we will attempt to include other imaging modalities to study the neovascularization of plaques with thickness < 2.5 mm. In addition, we included plaques with thickness ≥ 2.5 mm, which were considered high-risk plaques in a previous study. In future, we hope to obtain more information regarding plaques in high-risk groups and provide better management strategies.

## 5. Conclusion

The location and morphology of carotid plaques significantly associated with IPN grade on CEUS, and thereby carry the potential as biomarkers for plaque vulnerability. Serum HDL-C was further found as a protective factor against IPN, and may play a role in the management of carotid atherosclerosis. Our study not only provided a potential strategy for the identification of vulnerable carotid plaques, but also elucidated the important imaging predictors of stroke.

## Data availability statement

The raw data supporting the conclusions of this article will be made available by the authors, without undue reservation.

## Ethics statement

The studies involving human participants were reviewed and approved by Shanghai General hospital institutional review board. Written informed consent for participation was not required for this study in accordance with the national legislation and the institutional requirements.

## Author contributions

S-YG: conceptualization, writing–original draft, writing–review and editing, and investigation. L-NZ: investigation and writing–review and editing. FL: data curation. JC: writing–review and editing. M-HY: formal analysis and writing–review and editing. C-XJ: funding acquisition and writing–review and editing. RW: resources, funding acquisition, and writing–review and editing. All authors contributed to the article and approved the submitted version.

## Funding

This work was supported by the National Natural Science Foundation of China (grant numbers 82071931, 82130057, and 82202176), the Program for Shanghai Outstanding Medical Academic Leaders (grant number 2019LJ18), the Interdisciplinary Program of Shanghai Jiaotong University (grant number ZH2018ZDA17), and the Program from Science and Technology Commission of Shanghai Municipality (grant number 20Y11912400).

## Conflict of interest

The authors declare that the research was conducted in the absence of any commercial or financial relationships that could be construed as a potential conflict of interest.

## Publisher’s note

All claims expressed in this article are solely those of the authors and do not necessarily represent those of their affiliated organizations, or those of the publisher, the editors and the reviewers. Any product that may be evaluated in this article, or claim that may be made by its manufacturer, is not guaranteed or endorsed by the publisher.
