# Supplementary material for: Associations of plaque morphology and location with Intraplaque neovascularization in the carotid artery by contrast-enhanced ultrasound imaging
Source: Front Neurol. 2023 May 11;14:1097070. doi: 10.3389/fneur.2023.1097070 (PMC10213664; doi:10.3389/fneur.2023.1097070)
Supplement: Supplementary file 1 [file Table_1.DOCX]

Supplementary Material

# Supplementary Figures and Tables

## Supplementary Figures


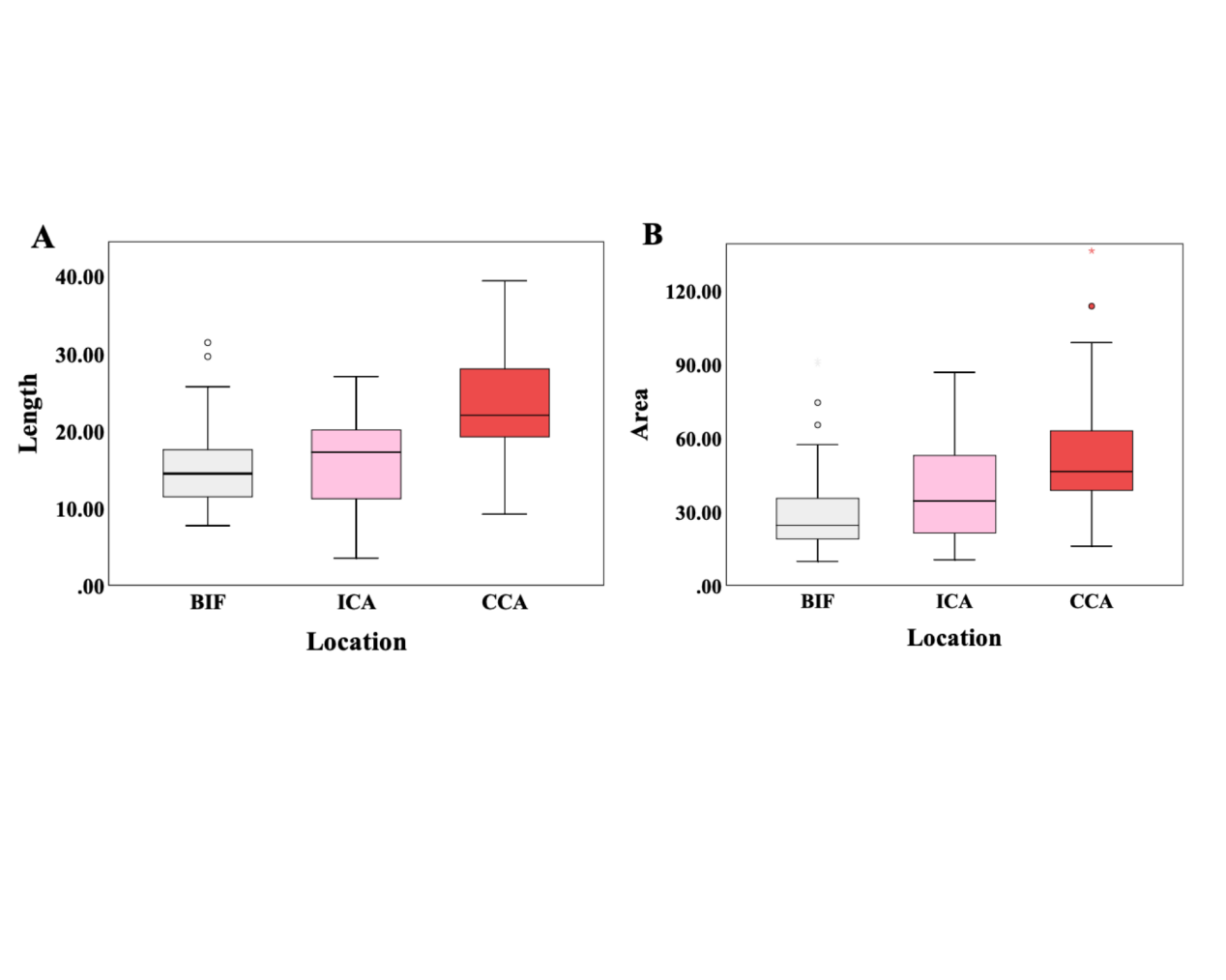


**Supplementary Figure 1.** Association between the size and location of plaque

**(A)** The plaques located in the CCA have a larger length; **(B)** The plaques located in the CCA have a larger area.

## Supplementary Tables

**Supplementary Table 1. Association between morphology, location and ulceration**

|  | **Without Ulceration** | **With Ulceration** | ***p*** |
| --- | --- | --- | --- |
| Carotid plaque morphology |  | | |
| Type I | 60 | 7 | .25 |
| Type II | 29 | 1 |  |
| Type III | 71 | 3 |  |
| Carotid plaque location |  |  |  |
| CCA | 103 | 5 | .13 |
| Carotid bifurcation | 22 | 4 |  |
| ICA | 35 | 2 |  |

**Supplementary Table 2. Association between the size and location of plaque**

|  | **BIF**  **(n = 108)** | **ICA**  **(n = 26)** | **CCA**  **(n = 37)** | ***p*** |
| --- | --- | --- | --- | --- |
| Length | 14.91 ± 7.49 | 16.38 ± 5.84 | 23.88 ± 7.98 | < .01^*^ |
| Thickness | 3.16 ± 0.82 | 3.65 ± 1.00 | 3.40 ± 0.62 | .10 |
| Area | 28.68 ± 14.85 | 39.11 ± 20.30 | 56.91 ± 32.31 | < .01^*^ |

**Supplementary Table 3. Association between blood lipid index and statin use history**

|  | **Stain use history**  **(n = 49)** | **Non stain use history**  **(n = 122)** | **OR** | **95%CI** | ***p*** |
| --- | --- | --- | --- | --- | --- |
| LDL-C, mmol/L | 2.56 ± 0.83 | 3.03 ± 0.81 | 0.89 | 0.63–1.26 | <.01^*^ |
| HDL-C, mmol/L | 1.19 ± 0.34 | 1.27 ± 0.36 | 0.33 | 0.13–0.81 | .19 |
| TC, mmol/L | 4.26 ± 1.15 | 4.86 ± 1.15 | 0.90 | 0.70–1.15 | <.01^*^ |
| TG, mmol/L | 1.53 ± 1.11 | 1.40 ± 0.08 | 1.20 | 0.86–1.68 | .45 |
